# Publication-ready single nucleotide polymorphism visualization with snipit

**DOI:** 10.1093/bioinformatics/btae510

**Published:** 2024-08-13

**Authors:** Áine O’Toole, Ammar Aziz, Daniel Maloney

**Affiliations:** Institute of Ecology and Evolution, University of Edinburgh, Edinburgh, EH93FL, United Kingdom; Victorian Infectious Diseases Reference Laboratory, The Royal Melbourne Hospital, Parkville, Victoria, VIC 3052, Australia; Institute of Ecology and Evolution, University of Edinburgh, Edinburgh, EH93FL, United Kingdom

## Abstract

**Summary:**

Snipit is an analysis and visualization tool designed for summarizing single nucleotide polymorphisms in sequences in comparison to a reference sequence. This tool efficiently catalogues nucleotide and amino acid differences, enabling clear comparisons through customizable, publication-ready figures. With features such as configurable colour palettes, customizable record sorting, and the ability to output figures in multiple formats, snipit offers a user-friendly interface for researchers across diverse disciplines. In addition, snipit includes a specialized *recombi-mode* for illustrating recombination patterns, which can highlight otherwise often difficult-to-detect relationships between sequences.

**Availability and implementation:**

Snipit is an open-source python-based tool that is hosted on GitHub under a GNU-GPL 3.0 licence (https://github.com/aineniamh/snipit). It can be installed from PyPi using pip. Source code and additional documentation can be found on the GitHub repository.

## 1 Main

Snipit is an analysis and visualization tool that summarizes single nucleotide polymorphisms (SNPs) relative to a reference sequence, enabling clear and fast comparisons between sequences of interest. When provided with an input alignment that includes a reference sequence, snipit will catalogue the differences (which can be output as a summary file) and produce a customizable, publication-ready figure in a variety of formats. The development of snipit was motivated by a need for comparing subtly different SARS-CoV-2 genome sequences to aid in outbreak investigations with the outbreak investigation software civet (O’Toole *et al.* 2022). However, the functionality of snipit has since been extended to include a variety of modes and customizable figure options. Although snipit has been useful in a number of SARS-CoV-2 studies—including outbreak investigations (Page *et al.* 2021, Hare *et al.* 2022), recombinant detection and analysis (Jackson *et al.* 2021, Wang *et al.* 2022, Wertheim *et al.* 2022, Tamura *et al.* 2023), and chronic infection investigations (Chaguza *et al.* 2023)—its use has since extended to other viral pathogens such as MPXV, HIV, Poliovirus, and Adenovirus (Isidro *et al.* 2022, Lambisia *et al.* 2023, Patiño *et al.* 2023, Serwin *et al.* 2023, O’Toole *et al.* 2024), and also to use in bacterial investigations including AMR detection (Martiny *et al.* 2022, Sim *et al.* 2022, 2023a,b). Snipit has the potential to be used for any investigation that requires comparison of SNPs against a reference genome and has already been used to help identify a number of new species of fungus (Ferretti *et al.* 2024a,b). We describe the main features of snipit below, how to access it and some potential use cases.

## 2 Features

### 2.1 Summarizing nucleotide differences relative to a reference sequence

**Figure 1.**
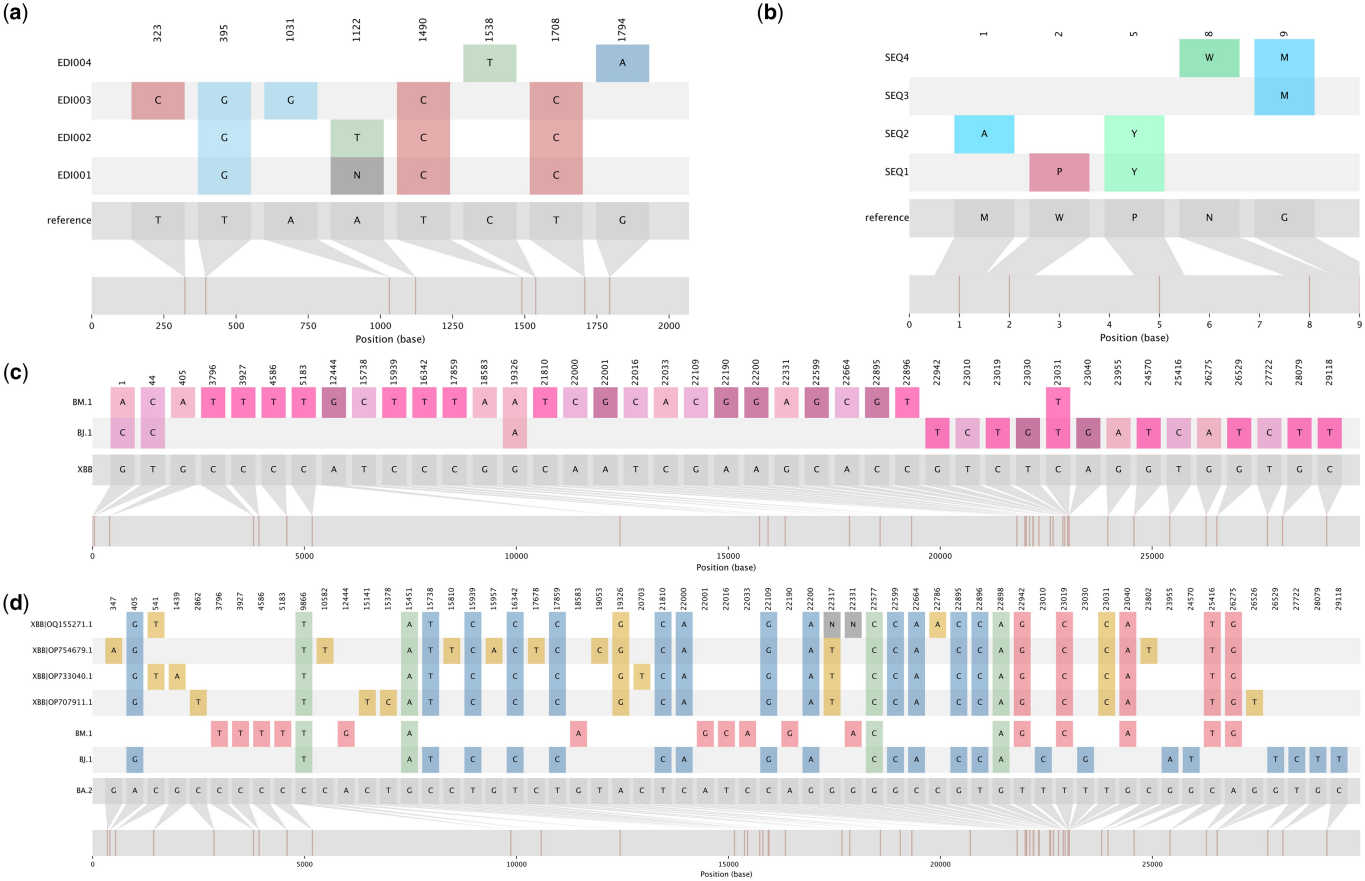
Snipit produces graphics that display SNP differences relative to a reference sequence in publication-ready figures. (a) With the default settings, snipit will assume the alignment provided is a nucleotide sequence and will produce a graph that colours SNPs by nucleotide change. Ambiguous changes are shown in grey. By default they do not create a new column in the snipit plot and only display if there are unambiguous changes at that site in other sequences. This can be configured with—ambig-mode. (b) Snipit can also display changes in an amino acid alignment relative to a reference amino acid sequence and colour palette can be configured to reflect the sequence type supplied. (c) Snipit has been widely used for recombination detection and visualization in viruses. Here the consensus sequences for three SARS-CoV-2 Pango lineages XBB, BJ.1 and BM.1 are shown in the plot, as in Tamura *et al.* (2023). XBB is a recombinant of BM.1 and BJ.1, and this can clearly be seen in the snipit plot, with different lineages contributing SNPs to either end of the XBB consensus sequence and the breakpoint likely to lie between positions 22896 and 22942 of the SARS-CoV-2 genome. (d) To further investigate recombinants, snipit has a dedicated recombi-mode that colours SNPs by whether they are common to both recombinant references specified (green), present in one or the other recombinant references (blue or red), or whether they are unique SNPs to a given query sequence (gold). In this case, the consensus sequence for BA.2 (the parent lineage of both BJ.1 and BM.1) is used as the overall reference sequence against which SNPs are detected, and BJ.1 and BM.1 are specified as the—recombi-references. Sequences of interest are then compared against these to aid recombinant detection. Consensus sequences are sourced from https://github.com/corneliusroemer/pango-sequences and example sequences of interest were sourced from GenBank (Accessions: OP707911.1, OP733040.1, OP754679.1, OQ155271.1).

In its default mode, snipit accepts a nucleotide multiple sequence alignment in FASTA format and catalogues the positions in the alignment that have unambiguous mutations relative to the reference sequence (i.e. a difference, i.e. A, C, G, or T, rather than an N or other ambiguity character). By default, the reference sequence is the first record in the alignment, however this can be configured with an optional flag to specify an alternative record identifier (-r/–reference). Any columns in the alignment with unambiguous mutations (A, C, G, or T) will be flagged, reported and visualized in the final figure. For each record in the alignment, all sites at the flagged columns are visualized and highlighted if they differ from the reference sequence, including any ambiguity character (Fig. 1a). The rationale behind only flagging unambiguously variable columns as the default stems from snipit’s original use case visualizing SARS-CoV-2 genome alignments, where amplicon dropouts are commonplace and there are many columns that vary ambiguously relative to the reference. We recognize that in some use cases all variable sites should be flagged and this is now a configurable option in snipit (—ambig-mode). With—ambig-mode all, any difference relative to the reference allele will be flagged and added to the visualization as an additional column. Mutations flagged and visualized can be output in a summary table in csv form with the—write-snps flag. The colours and other figure aesthetics, such as custom record sorting and vertical orientation of the plot, can be configured by the user. The figure can be output in png, jpg, tiff, pdf, or svg form. The vector svg output can be embedded in distributable html reports or webpages [as in civet and piranha (O’Toole *et al.* 2022, 2024)] or further modified to customize the figure for publication.

### 2.2 Summarizing amino acid differences relative to a reference sequence

Snipit has been extended to visualize amino acid alignments in addition to the original nucleotide implementation. This extends the tool, enabling it to now highlight functional changes between sequences relative to a reference starting point (Fig. 1b). To run an amino acid alignment through snipit, simply specify what sequence type (either aa for amino acid or nt for nucleotide) is being provided to the software with -t/—sequence-type. With this option, it is recommended to adjust the colour palette to one that encompasses amino acid information.

### 2.3 Recombination visualization using recombi-mode

Recombination can be challenging to detect using traditional phylogenetic methods, particularly between closely related populations. Snipit has a dedicated *recombi-mode* that facilitates visualization of recombination patterns. In *recombi-mode* additional references that represent the parent populations can be specified and mutations in the putative recombinant sequences are highlighted and colour-coded by whether they are shared between both parents, which parent if the mutation is unique to a given parent, or whether they are unique mutations not present in either parent population (Fig. 1c and d). Snipit produces an image that can demonstrate visually whether recombination is likely in the sequences of interest, as recombination is often characterized by runs of shared SNPs from one parent or the other.

## 3 Discussion

Here, we present snipit, a command-line tool that quickly visualizes mutations present in a multiple sequence alignment relative to a reference sequence. It is easy to install and the aesthetics of the final figure can be configured with a variety of options. Although the origins of the tool lie with visualization of SNPs and recombination in SARS-CoV-2, it has since been used to visualize a variety of other viruses, bacteria and fungi. Although snipit is agnostic to sequence origin and can accept any multiple sequence alignment, very large alignments with many changes (>100) become difficult to visualize effectively on a single plot. In this instance, we would recommend visualizing a region of interest in the alignment and perhaps using the—remove-site-text flag to prevent overlapping annotations in the figure. The user can specify custom widths and heights to ensure all SNPs are plotted, however snipit is used most effectively to highlight a number of key changes relative to reference. Popular genome browsers such as UCSC Genome Browser (Kent *et al.* 2002), the Ensembl Genome Browser (Hubbard *et al.* 2005) and the NCBI Genome Map Viewer (Wheeler *et al.* 2005) have much broader toolkits and for dynamic visualization of larger genomes may be more suitable resources. However, snipit can quickly highlight key residues and summarize changes relative to a reference sequence, producing publication-ready visualizations in a single step.
